# Efficacy of single‐pill combination in uncontrolled essential hypertension: A systematic review and network meta‐analysis

**DOI:** 10.1002/clc.24082

**Published:** 2023-07-11

**Authors:** Mengxin Xie, Tianjiao Tang, Hongsheng Liang

**Affiliations:** ^1^ Department of Cardiology Dongguan Children's Hospital Affiliated to Guangdong Medical University Shilong Dongguan China

**Keywords:** control rate, essential hypertension, network meta‐analysis, single‐pill combination

## Abstract

This study aimed to evaluate the efficacy of single‐pill combination (SPC) antihypertensive drugs in patients with uncontrolled essential hypertension. Through Searching Pubmed, EMBASE, the Cochrane Library, and Web of Science collected only randomized controlled trials on the efficacy of single‐pill combination antihypertensive drugs in people with uncontrolled essential hypertension. The search period is from the establishment of the database to July 2022. The methodological quality of the included studies was assessed using the Cochrane Risk of Bias Assessment, and statistical analyses were performed using Review Manage 5.3 and Stata 15.1 software. This review ultimately included 32 references involving 16 273 patients with uncontrolled essential hypertension. The results of the network meta‐analysis showed that a total of 11 single‐pill combination antihypertensive drugs were included, namely: Amlodipine/valsartan, Telmisartan/amlodipine, Losartan/HCTZ, Candesartan/HCTZ, Amlodipine/benazepril, Telmisartan/HCTZ, Valsartan/HCTZ, Irbesartan/amlodipine, Amlodipine/losartan, Irbesartan/HCTZ, and Perindopril/amlodipine. According to SUCRA, Irbesartan/amlodipine may rank first in reducing systolic blood pressure (SUCRA: 92.2%); Amlodipine/losartan may rank first in reducing diastolic blood pressure (SUCRA: 95.1%); Telmisartan/amlodipine may rank first in blood pressure control rates (SUCRA: 83.5%); Amlodipine/losartan probably ranks first in diastolic response rate (SUCRA: 84.5%). Based on Ranking Plot of the Network, we can conclude that single‐pill combination antihypertensive drugs are superior to monotherapy, and ARB/CCB combination has better advantages than other SPC in terms of systolic blood pressure, diastolic blood pressure, blood pressure control rate, and diastolic response rate. However, due to the small number of some drug studies, the lack of relevant studies has led to not being included in this study, which may impact the results, and readers should interpret the results with caution.

## Graphical abstract

High blood pressure significantly increases the incidence of stroke, myocardial infarction, heart failure, and chronic kidney disease and is the leading cause of cardiovascular disease and premature death worldwide. Single‐pill combination preparations are a hot topic in the field of treatment of essential hypertension. In this study, the efficacy of different single‐pill combinations (SPCs) in the treatment of patients with uncontrolled essential hypertension was evaluated by network meta‐analysis, and the optimal treatment regimen was selected according to SUCRA values, and the results showed that the angiotensin II receptor blocker (ARB)/calcium‐channel blockers (CCB) combination had a significant advantage in lowering blood pressure.

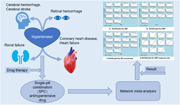

## 1 INTRODUCTION

Hypertension is defined as systolic blood pressure (SBP) values ≥140 mmHg and/or diastolic blood pressure (DBP) values ≥90 mmHg.^1^ In most people with hypertension, there is no clear cause, called essential hypertension, which accounts for more than 90% of all people with hypertension.^2^ High blood pressure significantly increases the incidence of stroke, myocardial infarction, heart failure, and chronic kidney disease and is the leading cause of cardiovascular disease and premature death worldwide.^3^ In 2019, approximately 1.2 billion people globally were estimated to have hypertension—twice as many as in the year 1990,^4^ Particularly in low‐ and middle‐income countries, prevalence is higher, severely increasing the burden and cost of the disease to global health systems. Preventing and controlling hypertension is a major global public health strategy for reducing premature mortality from cardiovascular disease (CVD).^5^ Therefore, it is essential to control high blood pressure. However, the overall global hypertension control rate remains low.^4^ Studies have shown that patients with treated but uncontrolled hypertension have a significantly increased risk of all‐cause mortality and cardiovascular mortality, so increasing the rate of blood pressure control may reduce the incidence of related complications.^6^

At present, a growing number of national guidelines for the management of hypertension recommend the use of single‐pill combination (SPC) for the treatment of hypertension.^7^, ^8^, ^9^ SPC is a compound tablet made by combining two or more drugs with different mechanisms of action in the same pill, which can achieve a hypotensive effect by synergizing with different antihypertensive mechanisms to use the lowest dose to reduce the incidence of adverse reactions and improve patient compliance. The common combinations used worldwide in the form of SPC for the treatment of hypertension are: Calcium‐channel blockers (CCB) + β‐receptor blockers, Diuretics + calcium channel blockers, β‐receptor blockers + angiotensin‐converting enzyme inhibitors (ACEI), angiotensin II receptor blocker (ARB) + diuretic, calcium channel blocker + ACEI/ARB, and other combinations.^10^ The study found that hypertensive patients who started an SPC had higher compliance after 1 year of treatment than monotherapy‐free combination.^11^ SPC may further improve blood pressure control rates by increasing patient compliance. Ahn Y^12^ compared an SPC amlodipine orotate/valsartan (AML/VAL) 5/160 mg with valsartan/hydrochlorothiazide (VAL/HCTZ) 160/12.5 mg in patients with uncontrolled essential hypertension with monotherapy and found that the AML/VAL group had a higher rate of blood pressure control than the VAL/HCTZ group after 8 weeks of treatment (84.3% V71.3%), and uric acid levels decreased in the AML/VAL group, while uric acid levels in the VAL/HCTZ group increased significantly. Another study comparing telmisartan/amlodipine with telmisartan/hydrochlorothiazide for essential hypertension showed similar rates of blood pressure control in both groups (63.9% v61.5), but the telmisartan/amlodipine group exerted a faster blood pressure lowering effect.^13^ Therefore, there is still a lack of evidence‐based recommendations on which SPC therapy is more effective in lowering blood pressure. Based on the network meta‐analysis, this study compared the efficacy of different kinds of SPC in patients with uncontrolled hypertension and ranked them so as to provide evidence‐based medical evidence for clinical medication.

Network meta‐analysis is a technique that combines direct and indirect evidence in a network of randomized controlled trials, compares the impact of multiple interventions simultaneously in a single analysis, and estimates the sequencing of each intervention to select the best treatment option.^14^ Therefore, in this study, we used network meta‐analysis to compare the antihypertensive efficacy of different SPCs in patients with uncontrolled essential hypertension, select the best antihypertensive regimen, and provide evidence‐based recommendations to patients and physicians.

## 2 MATERIALS AND METHODS

The protocol reviewed for this review is registered with PROSPERO (CRD42023403134).

### 2.1 Search strategy

The researchers in this paper searched four electronic databases (Pubmed, EMBASE, the Cochrane Library, and Web of Science) by computer from creation to July 2022. The search strategy is built around the PICOS tool: (P) population: patients with uncontrolled essential hypertension; (I) Intervention: use of single‐tablet combination drugs; (C) Comparison group: monotherapy, free combination of blood pressure reduction; (O) Results: Antihypertensive effect of patients with essential hypertension included systolic blood pressure, diastolic blood pressure, blood pressure control rate, and diastolic response rate after treatment. (S) Study type: Randomized controlled trial. (S) Study type: Randomized controlled trial. The detailed search strategy is shown in Table S1 (Pubmed is used as an example).

### 2.2 Inclusion criteria

#### 2.2.1 Experimental design

All included studies were clinical randomized controlled trials (RCTs).

#### 2.2.2 Research object

Patients with essential hypertension with systolic blood pressure ≥140 mmHg and/or diastolic blood pressure ≥90 mmHg after at least 4 weeks of monotherapy or free combination therapy and adults older than 18 years.

#### 2.2.3 Interventions

The experimental group was given SPC antihypertensive drugs to treat essential hypertension, and the control group was given other antihypertensive drugs to treat essential hypertension.

#### 2.2.4 Outcome indicator

1) Change of SBP in the office after treatment;
2) The change value of DBP in the office after treatment;
3) BP control rate (SBP < 140 mmHg and DBP < 90 mmHg);
4) DBP response rate (patients with a mean sitting DBP < 90 mmHg at the end of treatment and/or a decrease in mean sitting DBP of 10 mmHg).

### 2.3 Exclusion criteria

1) Patients with secondary hypertension;
2) Nonclinical randomized controlled trials;
3) Literature with incomplete data or inability to obtain full text;
4) Guidelines, reviews, conferences, meta‐analyses, animal and cell experiments;

Repeat publications.

### 2.4 Study selection

Use the document management software EndNote X 9.0 to filter and exclude documents. Two review authors first screened the titles of replicates, nonrandomized controlled trials, review papers, conference papers, and meta‐analyses. Two review authors then read abstracts to identify included and excluded studies. Finally, the remaining literature was read in its entirety by two review authors and further determined for inclusion. In this process, the two researchers independently screened the literature and finally compared the remaining literature; If they are the same, they are eventually included, and if they are different, they are discussed and resolved by a third investigator.

### 2.5 Data extraction

Standardized and preselected data extraction tables were used to record data to be included in studies under the following headings: (1) author, (2) year of publication, (3) country, (4) study participants, (5) total sample size, (6) mean age, (7) intervention, and (8) study period.

### 2.6 Risk of bias

Two researchers independently assessed the risk of bias (ROB) in accordance with the Cochrane Handbook version 5.1.0 tool for assessing ROB in RCTs. The following seven domains were considered: (1) randomized sequence generation, (2) treatment allocation concealment, blinding of (3) participants and (4) personnel, (5) incomplete outcome data, (6) selective reporting, and (7) other sources of bias. Trials were categorized into three levels of ROB by the number of components for which high ROB potentially existed: high risk (five or more), moderate risk (three or four), and low risk (two or fewer).^15^

### 2.7 Data analysis

Statistical analysis using Stata 15.1 and Review Manager 5.3. According to the characteristics of the data type, the effect size that can reasonably reflect the overall data was selected. If the analysis data were continuous data, the effect size was the mean difference (MD). The odds ratio (OR) was used if the data were binary. Effect sizes were presented with 95% confidence intervals (CI).

We used Stata 15.1 and based on the PRISMA NMA instruction manual, to simulate chains using Markov chain Monte Carlo simulation chains in a Bayesian‐based framework for NMA aggregation and analysis.^16^, ^17^ We use the node method to quantify and prove the agreement between indirect and direct comparisons, calculated by instructions in the Stata software, and if the *p*‐value > .05, the consistency is verified.^18^

Stata software was used to present and describe the network diagrams of different single‐pill combination drugs in the treatment of essential hypertension. The intervention network map is an intuitive representation of the evidence base, where each node in the generated network map represents a different intervention and different control conditions, and the lines connecting the nodes represent direct head‐to‐head comparisons between interventions. The size of each node and the width of the connecting line are proportional to the number of studies.^19^ The probability values were summarized and reported as the area under the cumulative ranked SUCRA curve, which was 0 when the treatment effect was the worst and 1 when the treatment effect was the best; the greater the area under the SUCRA curve, the better the treatment effect.^19^, ^20^

## 3 RESULTS

### 3.1 Study selection

**Figure 1:**
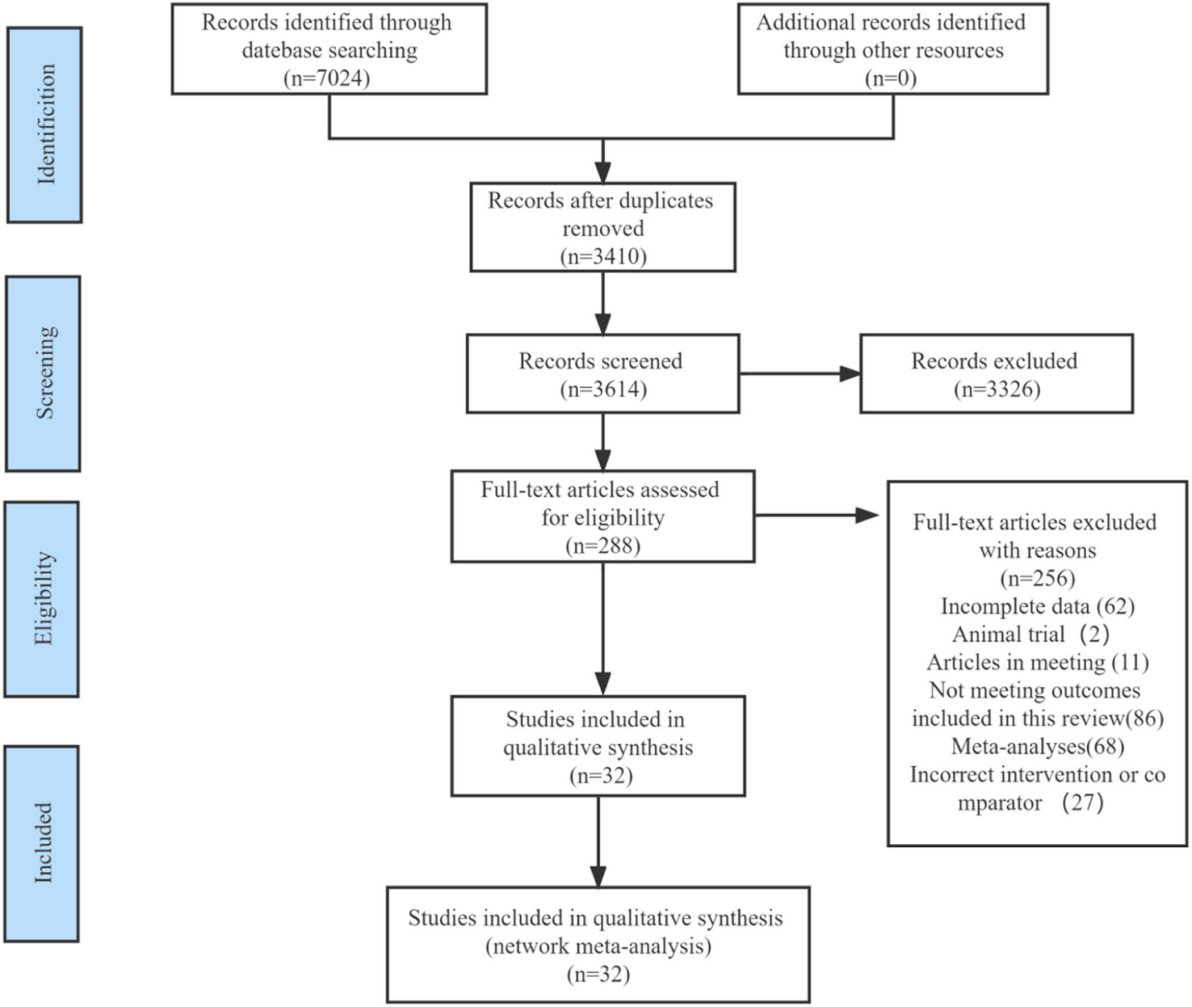
Flow diagram of literature selection.

The initial research resulted in 7024 references. After removing duplicates, reading the titles and abstracts of the remaining 3410 references, again excluding 3326 references after screening for potential studies, and searching the remaining 288 references in full text, of which 256 did not meet the eligibility criteria (reasons for exclusion included incomplete data, animal experiments, conference proceedings, failure to meet the results included in this study, incorrect interventions or comparisons, meta‐analyses). The last 32 studies were included in this study. After removing duplicates, the titles and abstracts of the remaining 3410 references were read. After screening for potential studies, 3326 references were again excluded, and the remaining 288 references were searched in full text. Of these, 256 did not meet the inclusion criteria (exclusion reasons included incomplete data, animal experiments, conference papers, failure to meet the results included in this study, incorrect intervention or comparison, and meta‐analysis). The final 32 studies were included in the meta‐analysis (Figure 1).

### 3.2 Risk of bias assessment

The risk of bias for included studies was evaluated with the Cochrane Risk of Bias Tool, and the results are shown in Figure S1. The included studies described random sequence generation, had no incomplete data, did not report selectively, and were assessed as low risk. Three studies showed allocation concealment and a low risk of bias. Twenty‐eight studies were blinded to assessors and were classified as at low risk of detection bias. Two studies showed additional bias due to small sample sizes and a high risk of bias.

### 3.3 Characteristics of the included studies

A total of 32 randomized controlled trials involving 16 273 people with uncontrolled essential hypertension were included. Eleven SPC were compared, namely Amlodipine/valsartan (8 studies),^12^, ^21^, ^22^, ^23^, ^24^, ^25^, ^26^, ^27^ Telmisartan/amlodipine (5 studies),^13^, ^28^, ^29^, ^30^, ^31^ Losartan/HCTZ (4 studies),^32^, ^33^, ^34^, ^35^ Candesartan/HCTZ (4 studies),^32^, ^36^, ^37^, ^38^ Amlodipine/benazepril (3 studies),^39^, ^40^, ^41^ Telmisartan/HCTZ (3 studies),^13,23,30^ Valsartan/HCTZ (3 studies),^12^, ^42^, ^43^ Irbesartan/amlodipine (2 studies),^44^, ^45^ Amlodipine/losartan (2 studies),^35^, ^46^ Irbesartan/HCTZ (1 studies),^47^ Perindopril/amlodipine (1 studies).^48^ The outcomes included were mainly systolic blood pressure, diastolic blood pressure, blood pressure control rate, and diastolic response rate after treatment. The characteristics of the included studies are shown in Table 1.

**Table 1:** Characteristics of the studies included in the meta‐analysis.

Author	Country	Year	Population	Age (mean = SD)	Total/male/female	Intervention	Intervention duration (week)	Outcome
Ohman^32^	Sweden	2000	Uncontrolled essential hypertension	EG*: ＜65 y (64.2%) ＞65 y (35.8%) CG*: ＜65 y (64.2%) ＞65 y (35.8%)	EG:151/81/70 CG:148/74/74	EG:Candesartan/HCTZ (16 mg/12.5 mg) CG:Losartan/HCTZ (50 mg/12.5 mg)	12	①②③④
Campbell^36^	UK	2001	Uncontrolled essential hypertension	EG:53.5 (9.0) CG:52.2 (9.2)	EG:164/101/63 CG:164/89/75	EG:Candesartan/HCTZ(16 mg/12.5 mg) CG:ARB (Candesartan 16 mg)	8	①②③④
Lacourciere^49^	Canada	2001	Uncontrolled essential hypertension	EG:55.6 (10.0) CG:55.0 (10.7)	EG:246/160/86 CG:245/150/95	EG:Telmisartan/HCTZ(80 mg/12.5 mg) CG:ARB (Telmisartan 80 mg)	8	①②③④
Mallion^42^	France	2003	Uncontrolled essential hypertension	EG:55.7 (11.2) CG:55.3 (11.2)	EG:666/353/313 CG:666/346/320	EG:Valsartan/HCTZ(160 mg/25 mg) CG:ARB (Valsartan 160 mg)	8	①②④
Chrysant^39^	USA	2004	Uncontrolled essential hypertension	EG:53.0 (10.0) CG:52.0 (11.0)	EG:164/92/72 CG:165/83/82	EG:Amlodipine/benazepril(10 mg/40 mg) CG:ACEI (Benazepril 40 mg)	8	①②④
Gleim^33^	USA	2006	Uncontrolled essential hypertension	EG:54.5 (11.0) CG:53.1 (9.4)	EG:147/85/62 CG:145/83/62	EG:Losartan/HCTZ(100 mg/12.5 mg) CG:ARB (Losartan 100 mg)	6	①②④
Chrysant^40^	USA	2007	Uncontrolled essential hypertension	EG:50.4 (10.5) CG:51.4 (10.6)	EG:273/151/122 CG:271/158/113	EG:Amlodipine/benazepril(10 mg/20 mg) CG:CCB (Amlodipine 10 mg)	6	①②④
Fogari^37^	Italy	2007	Uncontrolled essential hypertension	EG:55.0 (10.1) CG:55.3 (9.9)	EG:101/64/37 CG:102/52/50	EG:Candesartan/HCTZ(16 mg/12.5 mg) CG:CCB (Amlodipine 10 mg)	8	①②③④
Boenner^38^	Germany	2008	Uncontrolled essential hypertension	EG:54.6 (9.9) CG: 54.9 (10.2)	EG:648/381/267 CG:638/363/275	EG:Candesartan/HCTZ(32 mg/12.5 mg) CG:ARB (Candesartan 32 mg)	8	①②③④
Chen^50^	China	2008	Uncontrolled essential hypertension	EG:51.7 (9.4) CG:51.2 (9.6)	EG:175/105/70 CG:170/105/65	EG:Telmisartan/HCTZ(80 mg/12.5 mg) CG:ARB (Telmisartan 80 mg)	8	①②④
Tuomilehto^43^	Finland	2008	Uncontrolled essential hypertension	EG:53.9 (10.0) CG:54.2 (10.4)	EG:903/515/388 CG:899/512/387	EG:Valsartan/HCTZ(320 mg/12.5 mg) CG:ARB (320 mg)	8	①②③④
Neutel^47^	USA	2008	Uncontrolled essential hypertension	EG:55.1 CG:55.3	EG:328/181/147 CG:106/49/57	EG:Irbesartan/HCTZ(300 mg/25 mg) CG:ARB (Irbesartan 300 mg)	8	①②③
Schrader^21^	Germany	2009	Uncontrolled essential hypertension	EG:65.6 (7.56) CG:65.4 (7.16)	EG:592/307/285 CG:591/307/284	EG:Amlodipine/valsartan(5 mg/160 mg) CG:CCB (Amlodipine 10 mg)	8	①②③
Schunkert^22^	Germany	2009	Uncontrolled essential hypertension	EG:54.1 (12.0) CG:54.1 (12.2)	EG:473/251/222 CG:471/253/218	EG:Amlodipine/valsartan(10 mg/160 mg) CG:CCB (Amlodipine 10 mg)	8	①②④
Sinkiewicz^23^	Poland	2009	Uncontrolled essential hypertension	EG:55.4 CG:54.5	EG:322/174/148 CG:308/172/136	EG:Amlodipine/valsartan(5 mg/160 mg) CG:ARB (Valsartan 160 mg)	8	①②④
Ke^24^	China	2010	Uncontrolled essential hypertension	EG:53.4 (9.7) CG:54.2 (9.1)	EG:347/213/134 CG:349/240/109	EG:Amlodipine/valsartan(5 mg/80 mg) CG:CCB (Amlodipine 5 mg)	8	①②③④
Huang^25^	China	2011	Uncontrolled essential hypertension	EG:51.6 (10.8) CG:51.7 (8.9)	EG:308/181/127 CG:306/191/115	EG:Amlodipine/valsartan(5 mg/80 mg) CG:ARB (Valsartan 80 mg)	8	①②③④
Neldam^28^	Denmark	2011	Uncontrolled essential hypertension	EG:55.5 (9.8) CG:56.4 (10.4)	EG:317/171/146 CG:315/187/128	EG:Telmisartan/amlodipine(80 mg/10 mg) CG:CCB (Amlodipine 10 mg)	8	①②③④
Neldam^29^	Denmark	2011	Uncontrolled essential hypertension	EG:53.9 (11.0) CG:54.3 (10.6)	EG:277/160/117 CG:276/176/100	EG:Telmisartan/amlodipine(40 mg/5 mg) CG: CCB (Amlodipine 10 mg)	8	①②③④
Bobrie^44^	France	2012	Uncontrolled essential hypertension	EG:58.1 (11.0) CG:56.4 (11.4)	EG:144/69/75 CG:143/69/74	EG:Irbesartan/amlodipine (150 mg/10 mg) CG:CCB (Amlodipine 10 mg)	8	①②③
Bobrie^45^	France	2012	Uncontrolled essential hypertension	EG:56.0 (11.5) CG:57.4 (11.3)	EG:155/62/93 CG:165/70/95	EG:Irbesartan/amlodipine(300 mg/5 mg) CG:ARB (Irbesartan 300 mg)	8	①②③
Hong^46^	Korea	2012	Uncontrolled essential hypertension	EG:50.8 (9.9) CG:49.8 (10.9)	EG:70/49/21 CG:72/59/13	EG:Amlodipine/losartan(5 mg/100 mg) CG:ARB (losartan 100 mg)	8	①②④
Toh^34^	Japan	2012	Uncontrolled essential hypertension	EG:69.6 (9.6) CG:67.4 (11.6)	EG:98/60/38 CG:95/63/32	EG:Losartan/HCTZ(50 mg/12.5 mg) CG:ARB	12	①②③
Wang^26^	China	2013	Uncontrolled essential hypertension	EG:53.8 (8.6) CG:53.1 (8.7)	EG:272/137/135 CG:268/133/135	EG:Amlodipine/valsartan(5 mg/80 mg) CG:CCB (Nifedipine 30 mg)	12	①②③
Suh^35^	Korea	2014	Uncontrolled essential hypertension	EG:51. 2(10.1) CG:52.0 (9.9)	EG:97/67/30 CG:93/67/26	EG:Amlodipine/losartan(5 mg/100 mg) CG:Losartan/HCTZ(100 mg/12.5 mg)	8	①②③④
Yan^41^	China	2014	Uncontrolled essential hypertension	EG:50.7 (9.0) CG:51.8 (10.2)	EG:111/70/41 CG:114/83/31	EG:Amlodipine/benazepril(2.5 mg/10 mg) CG:ACEI (Benazepril 10 mg)	8	①②③
Ihm^30^	Korea	2016	Uncontrolled essential hypertension	EG:57.1 (10.5) CG:54.9 (8.5)	EG:63/43/20 CG:60/45/15	EG:Telmisartan/amlodipine(40 mg/2.5 mg) CG: CCB (S‐amlodipine 2.5 mg)	8	①②③④
Kondo^13^	Japan	2016	Uncontrolled essential hypertension	EG:64.3 (12.4) CG:68.7 (10.9)	EG:36/25/11 CG:39/24/15	EG:Telmisartan/amlodipine(40 mg/5 mg) CG:Telmisartan/HCTZ(40 mg/12.5 mg)	12	①②③④
Park^31^	Korea	2016	Uncontrolled essential hypertension	EG:53.5 (7.9) CG:53.1 (8.1)	EG:59/54/5 CG:59/51/8	EG:Telmisartan/amlodipine(40 mg/2.5 mg) CG:ARB (Telmisartan 80 mg)	8	①②③
Sung^27^	Korea	2016	Uncontrolled essential hypertension	EG:54.0 (11.0) CG:54.0 (11.0)	EG:110/35/75 CG:111/32/79	EG:Amlodipine/valsartan(5 mg/160 mg) CG:CCB (Amlodipine10mg)	8	①②③
Hu^48^	China	2016	Uncontrolled essential hypertension	EG:51.1 (10.1) CG1:52.5 (9.1) CG2:52.7 (9.0)	EG:247/139/108 CG1:245/135/110 CG2:175/91/84	EG:Perindopril/amlodipine(4 mg/5 mg) CG1:CCB (Amlodipine 5 mg) CG2:ACEI (Perindopril 4 mg)	8	①③④
Ahn^12^	Korea	2018	uncontrolled essential hypertension	EG:56.1 (10.0) CG:57.7 (11.3)	EG:121/90/31 CG:117/96/21	EG:Amlodipine/valsartan(5 mg/160 mg) CG:Valsartan/HCTZ (160 mg/12.5 mg)	8	①②③

*Note*: EG\CG*: The ages of the experimental and control groups were not reported separately in the study, only the age range was reported. ①: Systolic blood pressure; ②: Diastolic blood pressure; ③: Blood pressure control rate; ④: Diastolic blood pressure response rate.

Abbreviations: ACEI, angiotensin‐converting enzyme inhibitors; ARB, angiotensin receptor blockers; CCB, calcium channel blockers; CG, control group; EG, experimental group; HCTZ, hydrochlorothiazide.

### 3.4 Network Meta‐Analysis

#### 3.4.1 Network meta‐analysis of the efficacy of SBP

All *P*‐values for indirect and direct comparisons between all studies were tested for consistency and inconsistency, and *p*‐values were greater than .05, indicating that the effect of consistency between studies was acceptable. Details will be shown in Table S2.

**Figure 2:**
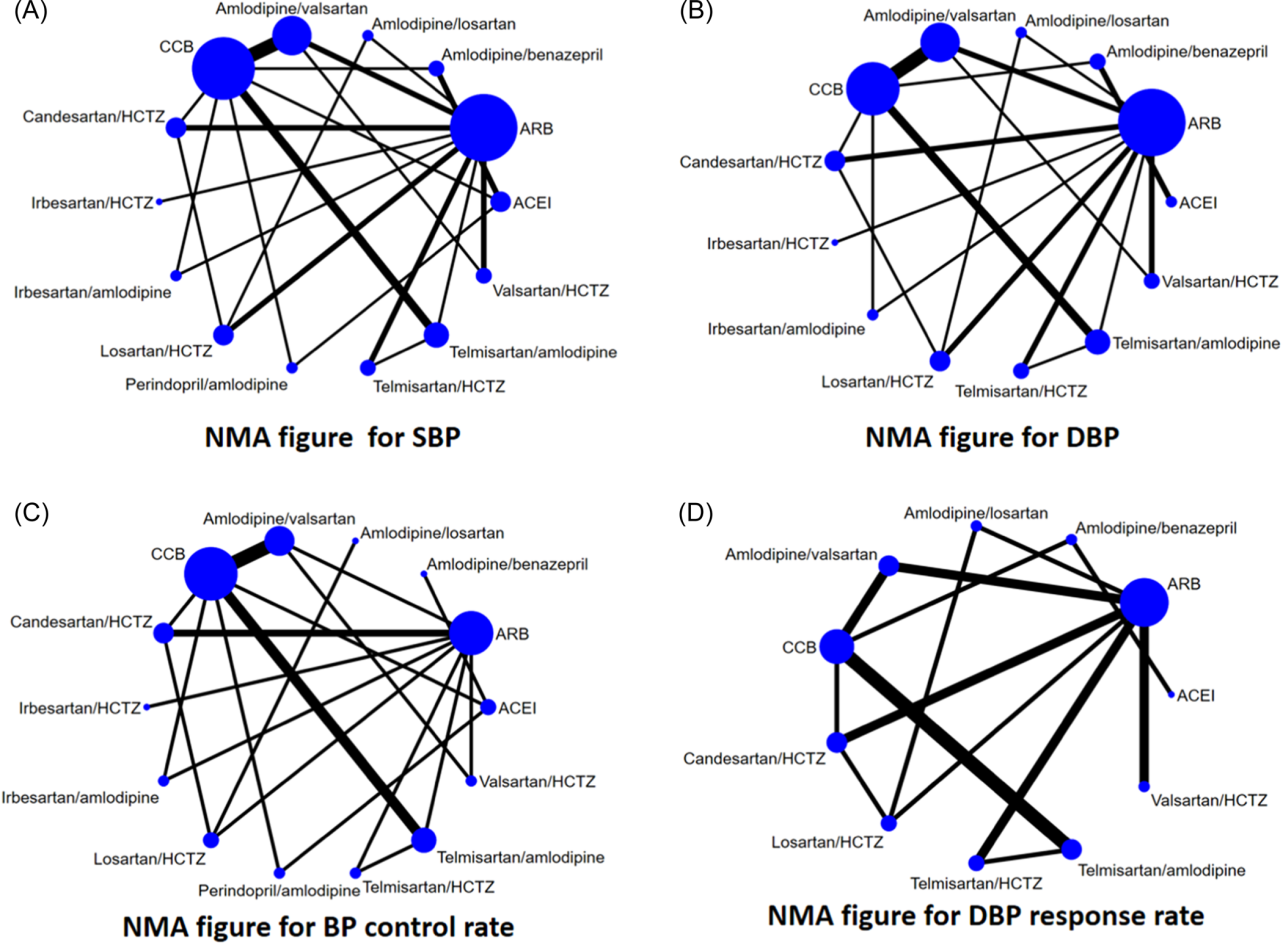
NMA figure for each outcome indicator. DBP, diastolic blood pressure; SBP, systolic blood pressure.

**Figure 3:**
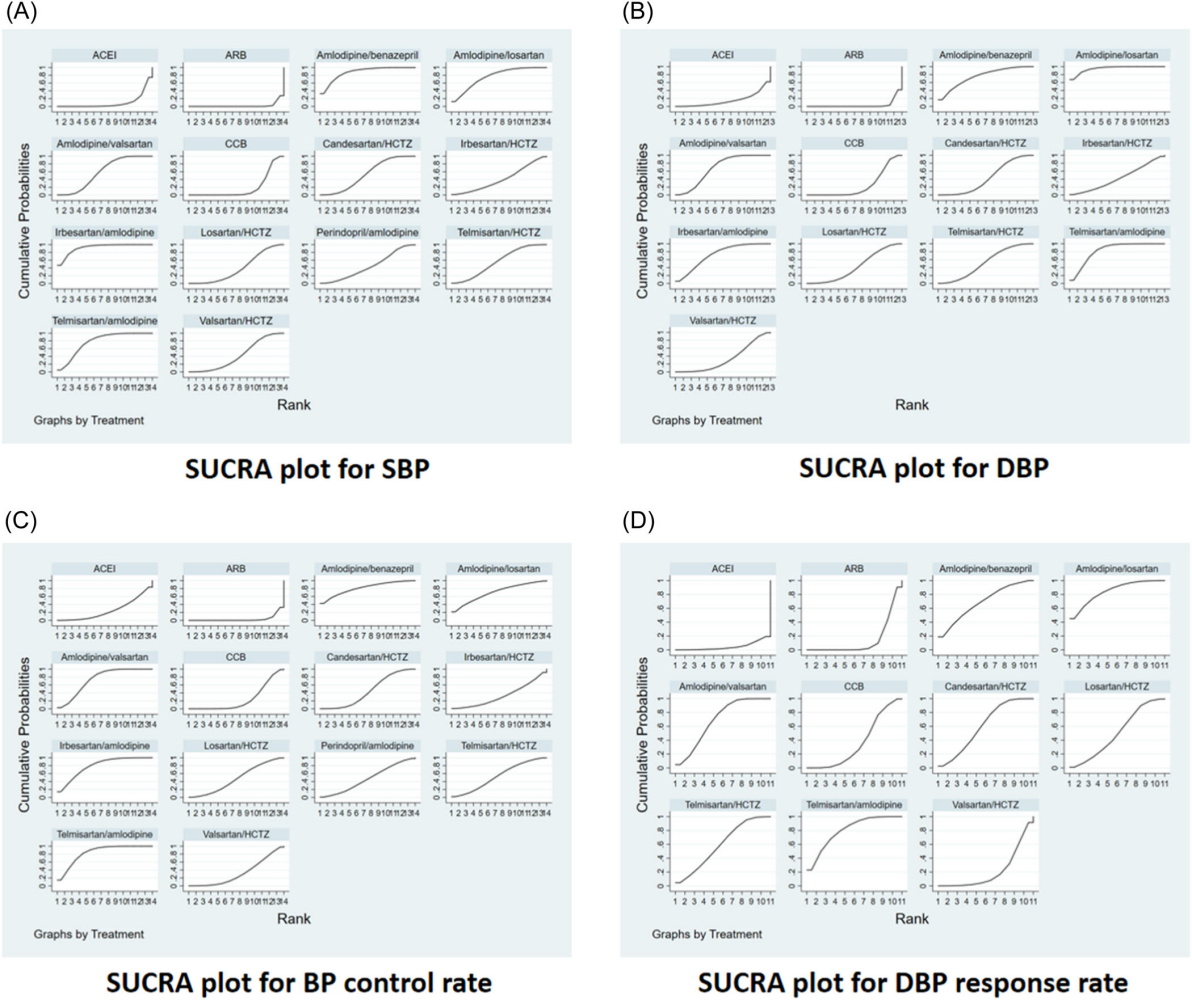
SUCRA values for each outcome indicator. DBP, diastolic blood pressure; SBP, systolic blood pressure.

In terms of systolic blood pressure, 32 studies covering 14 interventions were included, and the network map is shown in Figure 2A. Through statistical analysis of 14 interventions, a cumulative ranking probability plot was produced, and the corresponding SUCRA value was calculated to obtain treatment regimen ranking. The SUCRA curve area plot (Figure 3A) plotted by Stata 15.1 software was sorted as follows: Irbesartan/amlodipine (92.2%) > Amlodipine/benazepril (87.6%) > Telmisartan/amlodipine (77.4%) > Amlodipine/losartan (76.1%) > Amlodipine/valsartan (58.9%) > Telmisartan/HCTZ (55.6%) > Candesartan/HCTZ (53.5%) > Perindopril/amlodipine (44.1%) > Valsartan/HCTZ（42.5%) > Irbesartan/HCTZ (40.9%) > Losartan/HCTZ（39.4%) > CCB (19.5%) > ACEI (10.0%) > ARB (2.3%). Table S6 shows a plot of direct or indirect analysis of the efficacy of 14 interventions in treating patients with uncontrolled essential hypertension. The blue shaded part indicates that the difference is significant. Irbesartan/amlodipine ranked first in the efficacy of systolic blood pressure reduction by sorting the SUCRA curve area chart.

#### 3.4.2 Network meta‐analysis of the efficacy of DBP

All *P*‐values for indirect and direct comparisons between all studies were tested for consistency and inconsistency, and all *p*‐values were greater than .05, indicating that the effect of consistency between studies was acceptable. Details will be shown in Table S3.

For diastolic blood pressure, 31 studies involving 13 interventions were included, and the network map is shown in Figure 2B. Through statistical analysis of 13 interventions, a cumulative ranking probability plot was produced, and the corresponding SUCRA value was calculated to obtain treatment regimen ranking. The SUCRA curve area plot (Figure 3B) plotted by Stata 15.1 software yielded the following rankings: Amlodipine/losartan (95.1%) > Telmisartan/amlodipine (82.2%) > Amlodipine/Benazepril (75.2%) > Irbesartan/Amlodipine (72.3%) > Amlodipine/valsartan (67.4%) > Telmisartan/HCTZ (52.2%) > Candesartan/HCTZ (43.2%) > Irbesartan/HCTZ (43.0%) > Losartan/HCTZ (42.6%) >Valsartan/HCTZ (33.5%) > CCB (24.6%) > ACEI (14.8%) > ARB (3.6%). Table S7 shows a plot of direct or indirect analysis of the efficacy of 13 interventions in reducing blood pressure in patients with uncontrolled essential hypertension. The blue shaded part indicates that the difference is significant. Amlodipine/losartan ranked first in reducing diastolic blood pressure by sorting the SUCRA curve area chart.

#### 3.4.3 Network meta‐analysis of the efficacy of blood pressure (BP) control rate

All *p*‐values for indirect and direct comparisons between all studies were tested for consistency and inconsistency, and all *p*‐values were greater than .05, indicating that the effect of consistency between studies was acceptable. Details will be shown in Table S4.

Regarding BP control rate, 24 studies involving 14 interventions were included, and the network map is shown in Figure 2C. Through statistical analysis of 14 interventions, a cumulative ranking probability plot was produced, and the corresponding SUCRA value was calculated to obtain treatment regimen ranking. The SUCRA curve area plot (Figure 3C) plotted by Stata 15.1 software was sorted as follows: Telmisartan/amlodipine (83.5%) > Amlodipine/Benazeprinil (81.3%) > Irbesartan/amlodipine (79.4%) > Amlodipine/valsartan (73.8%) > Amlodipine/losartan (69.7%) > Telmisartan/HCTZ (53.7%) > Losartan/HCTZ (47.5%) > Perindopril/amlodipine (47.2%) > Candesartan/HCTZ (44.9%) > Irbesartan/HCTZ (32.9%) > Valsartan/HCTZ (32.0%) > ACEI (25.6%) > CCB (24.9%) > ARB (3.5%). Table S8 shows a direct or indirect analysis of the antihypertensive efficacy of 14 interventions in patients with uncontrolled essential hypertension. The blue shaded part indicates that the difference is significant. Telmisartan/amlodipine ranked first in BP control rate by sorting the SUCRA curve area chart.

#### 3.4.4 Network meta‐analysis of the efficacy of DBP response rate

All *p*‐values for indirect and direct comparisons between all studies were tested for consistency and inconsistency, and all *p*‐values were greater than .05, indicating that the effect of consistency between studies was acceptable. Details will be shown in Table S5.

Regarding the DBP response rate, 22 studies involving 11 interventions were included, and the network map is shown in Figure 2D. Through statistical analysis of 14 interventions, a cumulative ranking probability plot was produced, and the corresponding SUCRA value was calculated to obtain treatment regimen ranking. The SUCRA curve area plot (Figure 3D) plotted by Stata 15.1 software resulted in the following order: Amlodipine/losartan (84.5%) > Telmisartan/amlodipine (79.9%) > Amlodipine/valsartan (69.0%) > Amlodipine/Benazepril (68.1%) > Candesartan/HCTZ (60.7%) > Telmisartan/HCTZ (59.6%) > Losartan/HCTZ (51.3%) > CCB (36.0%) > Valsartan/HCTZ (21.3%) > ARB (14.9%) > ACEI (4.6%). Table S9 shows a direct or indirect analysis of the efficacy of 14 interventions in patients with uncontrolled primary hypertension. The blue shaded part indicates that the difference is significant. Sorted by the SUCRA curve area chart, Amlodipine/losartan ranked first in DBP response rate.

### 3.5 Publication bias test

**Figure 4:**
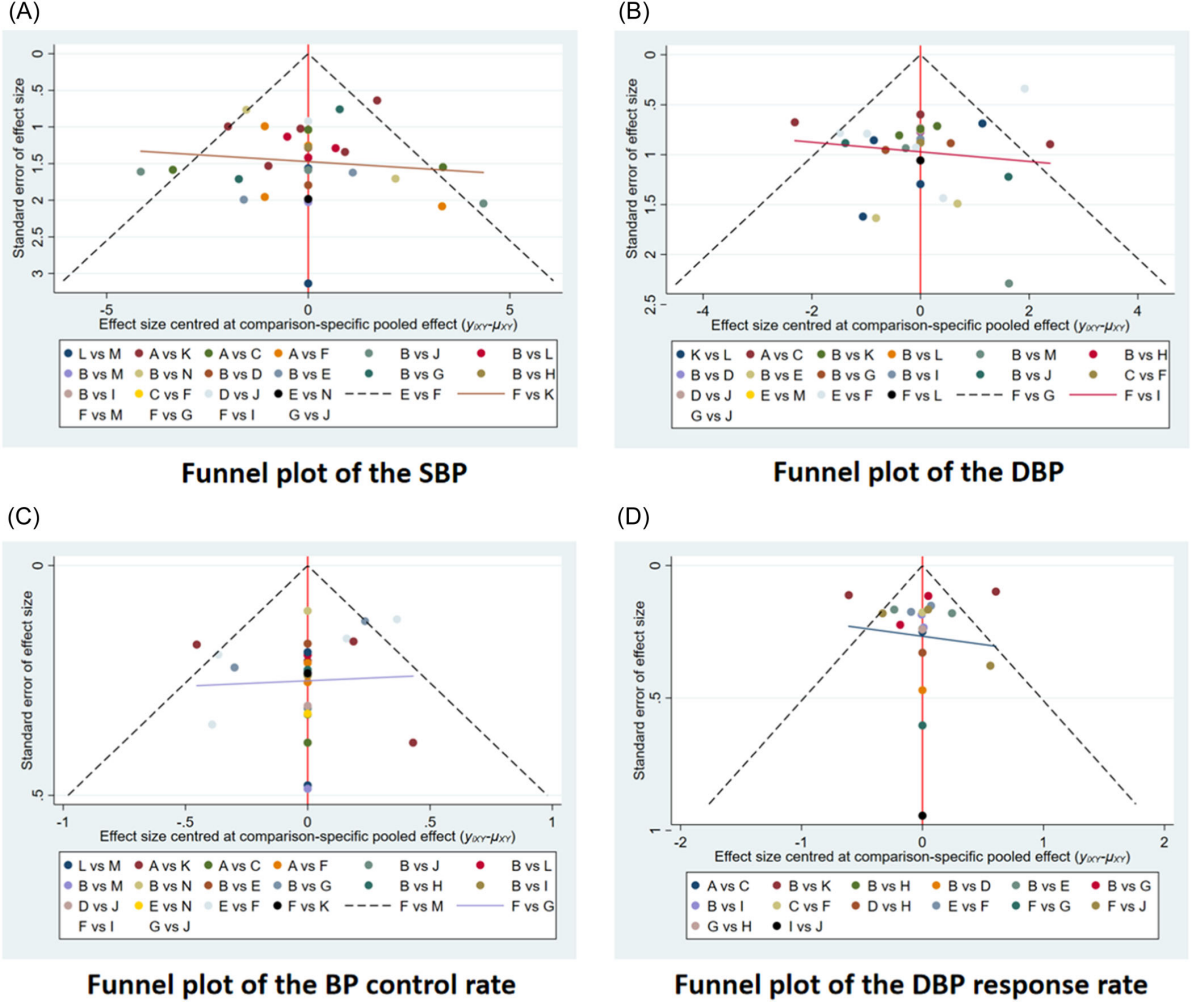
Funnel plots for each outcome indicator. DBP, diastolic blood pressure; SBP, systolic blood pressure.

We constructed separate funnel plots for all outcome measures to test for possible publication bias. From the funnel plot, we can see that the points on both sides of the line are basically symmetrical, and we did not find any significant publication bias. Funnel plots of posttreatment SBP, posttreatment DBP, BP control rate, and DBP response rate are shown in Figure 4.

## 4 DISCUSSION

In this review, we compared the antihypertensive efficacy of different SPC drugs in people with essential hypertension whose blood pressure remains uncontrolled despite monotherapy. A total of 32 randomized controlled trials, including 11 different SPC, were included, including 16 273 patients with uncontrolled essential hypertension, which is a considerable sample size. Our study shows that SPC drugs are better than monotherapy in reducing systolic and diastolic blood pressure and improving blood pressure control. Among them, in terms of systolic blood pressure, Irbesartan/amlodipine drug treatment has the best effect. In terms of diastolic blood pressure and diastolic blood pressure response rate, Amlodipine/losartan drug treatment has the best effect, and Telmisartan/amlodipine drug treatment has the best effect on blood pressure control rate. Overall, we believe that ARB/CCB combination formulations have the best antihypertensive effect.

Essential hypertension is characterized by slow onset and lack of characteristic symptoms, mainly by higher‐than‐normal blood pressure. The drug treatment of essential hypertension has many schemes. Most hypertensive patients need to use two or more drugs to achieve the target value of blood pressure.^51^ Increasing the type of drug will increase the patient's drug burden so that the patient's medication compliance decreases, and SPC combines two drugs with different mechanisms of action, which can improve the patient's compliance by reducing the number of medications.^52^ In a recent meta‐analysis,^53^ investigating the efficacy of a single‐pill triple combination for the treatment of uncontrolled hypertension, the triple combination reduced systolic blood pressure by an average of 24 mmHg from baseline and diastolic blood pressure by an average of 12 mmHg. For patients with hypertension, simplifying the treatment regimens can improve patient adherence. Adherence is a key factor in improving the rate of blood pressure control, and poor adherence can lead to decreased blood pressure control, increased complications of hypertension, increased number of clinical visits, and increased costs of hypertension management.^54^ A large retrospective study^55^ found that treatment with SPC reduced all‐cause mortality, the incidence of cardiovascular events, improved patient adherence with medications, and lasted longer for the drugs in the SPC group than in the combination group, compared with multidrug combination therapy. A meta‐analysis^56^ of 44 studies showed that SPC treatment improved adherence and persistence, further improving blood pressure control.

Among the many SPC drugs, the combination of calcium channel blockers and renin‐angiotensin system inhibitors (RASI), either angiotensin‐converting enzyme inhibitors (ACEIs) or angiotensin receptor blockers (ARBs), has been shown to be effective and safe in the treatment of patients with hypertension.^57^, ^58^ In addition, some patients with hypertension will be accompanied by hyperlipidemia, Jo et al.^59^ found that olmesartan/amlodipine/rosuvastatin combination treatment for patients with both hypertension and dyslipidemia is safe and effective in reducing blood pressure and LDL‐C. Therefore, this combination can provide an effective treatment option for patients with hypertension and hyperlipidemia.

Overall, our study has some clinical significance. First of all, SPC antihypertensive drugs have a significant effect on the treatment of essential hypertension. Secondly, when monotherapy is less effective in the treatment of essential hypertension, SPC preparation can be chosen, and combined with our findings, the ARB/CCB combination is the best combination.

## 5 STRENGTHS AND LIMITATIONS

Our study is the first network meta‐analysis comparing the efficacy of SPC in people with uncontrolled essential hypertension. The quality of randomized controlled trials included in this review was generally high, including 32 studies with 16 273 patients with uncontrolled essential hypertension, which is a very large sample size, and we tried to include as many studies as possible to compare the efficacy of different types of SPC, which provides newer and more comprehensive evidence‐based recommendations.

But our study shares some limitations with the studies on which it is based. First, There are many types of SPC, some drugs were not involved, and the lack of relevant randomized controlled trials had some impact on the results. Second, differences in patient population, baseline clinical value, drug dose, and duration of treatment across all RCTs may have influenced the results. In addition, some of our studies are small in number, and evidence for direct comparisons of some interventions is limited. Readers should interpret the results with caution, and future studies will need to be supplemented with more large and high‐quality randomized controlled trials. In conclusion, due to the inherent complexity of SPC, including the dose of SPC, the combination of SPC, and the lack of relevant literature and sample sizes, more relevant studies are needed to demonstrate this result. It also highlights the need for further expansion of relevant studies.

## 6 CONCLUSIONS

In our study, we recommend the use of SPC antihypertensive drugs for blood pressure control, and the ARB/CCB combination is preferred. However, the data from this study and the results of previous clinical trials demonstrate the efficacy of SPC in the treatment of uncontrolled essential hypertension and support the recommendation that for patients who do not achieve target blood pressure with low‐dose antihypertensives, SPC selection may reach the target blood pressure more quickly than increasing the dose of monotherapy. Due to the large variety of SPC drugs, high‐quality randomized controlled trials with large sample sizes are still needed to evaluate the efficacy of single‐tablet combination preparations in the treatment of hypertension. Readers should interpret the results with caution.

## AUTHOR CONTRIBUTIONS

Mengxin Xie interpreted the data, wrote the initial manuscript, and was involved in the data analysis. Tianjiao Tang was responsible for the collection of all relevant papers. Hongsheng Liang was responsible for the supervision of the study. Both authors have read and agreed to the published version of the manuscript.

## CONFLICTS OF INTEREST STATEMENT

The authors declare no conflict of interest.

## Supporting information

Supporting information.

Supporting information.

## Data Availability

The data that support the findings of the study are available fromthe first author, upon reasonable request
