# Supplementary material for: Efficacy of single‐pill combination in uncontrolled essential hypertension: A systematic review and network meta‐analysis
Source: Clin Cardiol. 2023 Jul 11;46(8):886–98. doi: 10.1002/clc.24082 (PMC10436803; doi:10.1002/clc.24082)
Supplement: Supplementary file 1 — Supporting information. [file CLC-46-886-s001.pdf]

Supplementary

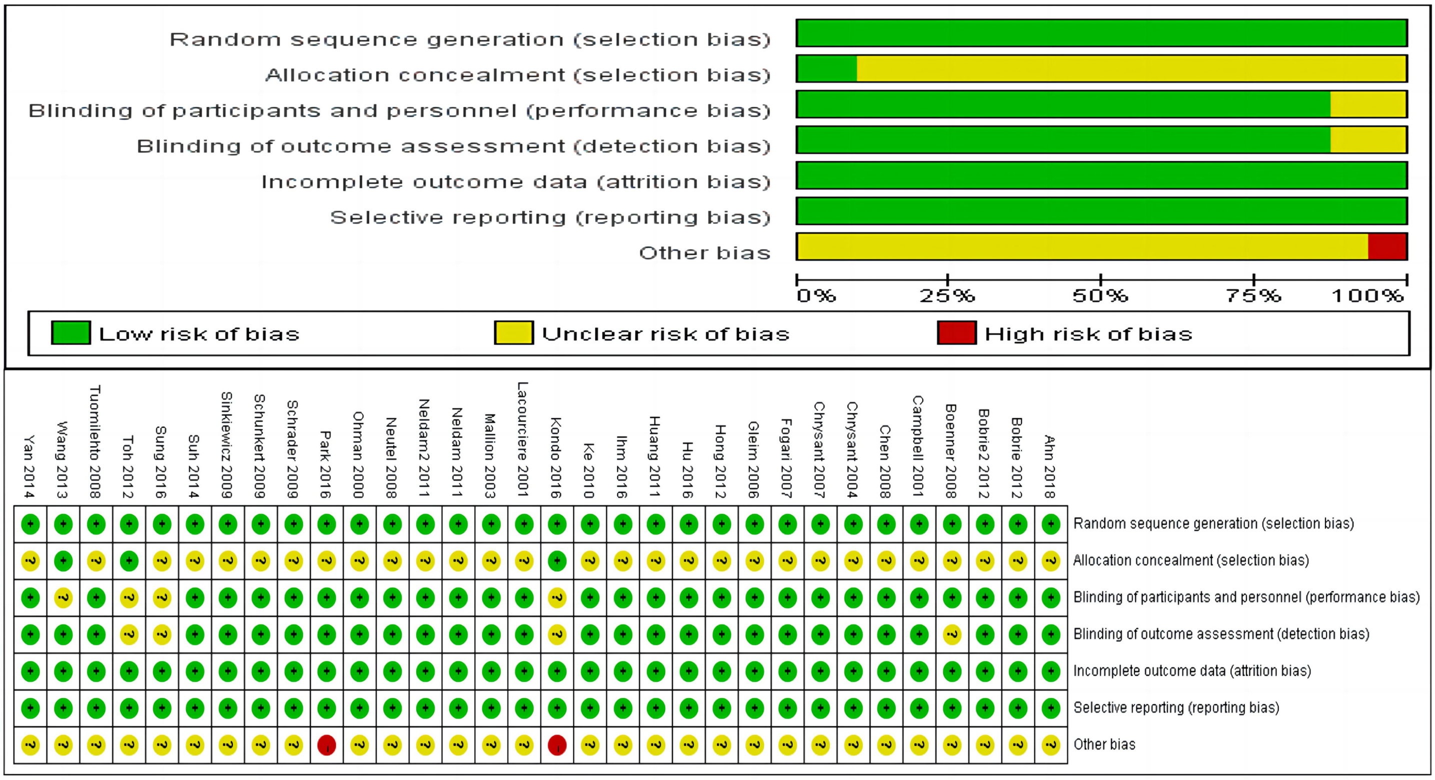

Supplementary Figure 1. Analysis of the risk of bias in accordance with the Cochrane collaboration guideline.
