# Supplementary material for: Efficacy of single‐pill combination in uncontrolled essential hypertension: A systematic review and network meta‐analysis
Source: Clin Cardiol. 2023 Jul 11;46(8):886–98. doi: 10.1002/clc.24082 (PMC10436803; doi:10.1002/clc.24082)
Supplement: Supplementary file 2 — Supporting information. [file CLC-46-886-s002.docx]

Table S1. Search strategy on PubMed

| #1 | "Essential Hypertension"[Mesh] |
| --- | --- |
| #2 | ((primary hypertension[Title/Abstract]) OR (high blood pressure[Title/Abstract])) OR (hypertension[Title/Abstract]) |
| #3 | #1 OR #2 |
| #4 | Single Pill Combination[Title/Abstract] |
| #5 | ((((((((((((((((((((((((((((((((((((((((((((((((((((((((((((((fixed-dose combination[Title/Abstract]) OR (SPC[Title/Abstract])) OR (FDC[Title/Abstract])) OR (Benazepril/Hydrochlorothiazide[Title/Abstract])) OR (Benazepril[Title/Abstract] AND Hydrochlorothiazide[Title/Abstract])) OR (Captopril/Hydrochlorothiazide[Title/Abstract])) OR (Captopril[Title/Abstract] AND Hydrochlorothiazide[Title/Abstract])) OR (Enalapril/Hydrochlorothiazide[Title/Abstract])) OR (Enalapril[Title/Abstract] AND Hydrochlorothiazide[Title/Abstract])) OR (Fosinopril/Hydrochlorothiazide[Title/Abstract])) OR (Fosinopril[Title/Abstract] AND Hydrochlorothiazide[Title/Abstract])) OR (Lisinopril/Hydrochlorothiazide[Title/Abstract])) OR (Lisinopril[Title/Abstract] AND Hydrochlorothiazide[Title/Abstract])) OR (Moexipril/Hydrochlorothiazide[Title/Abstract])) OR (Moexipril[Title/Abstract] AND Hydrochlorothiazide[Title/Abstract])) OR (Quinapril/Hydrochlorothiazide[Title/Abstract])) OR (Quinapril[Title/Abstract] AND Hydrochlorothiazide[Title/Abstract])) OR (Azilsartan/Chlorthalidone[Title/Abstract])) OR (Azilsartan[Title/Abstract] AND Chlorthalidone[Title/Abstract])) OR (Candesartan/Hydrochlorothiazide[Title/Abstract])) OR (Candesartan[Title/Abstract] AND Hydrochlorothiazide[Title/Abstract])) OR (Eprosartan/Hydrochlorothiazide[Title/Abstract])) OR (Eprosartan[Title/Abstract] AND Hydrochlorothiazide[Title/Abstract])) OR (Irbesartan/Hydrochlorothiazide[Title/Abstract])) OR (Irbesartan[Title/Abstract] AND Hydrochlorothiazide[Title/Abstract])) OR (Losartan/Hydrochlorothiazide[Title/Abstract])) OR (Losartan[Title/Abstract] AND Hydrochlorothiazide[Title/Abstract])) OR (Olmesartan/Hydrochlorothiazide[Title/Abstract])) OR (Olmesartan[Title/Abstract] AND Hydrochlorothiazide[Title/Abstract])) OR (Telmisartan/Hydrochlorothiazide[Title/Abstract])) OR (Telmisartan[Title/Abstract] AND Hydrochlorothiazide[Title/Abstract])) OR (Valsartan/Hydrochlorothiazide[Title/Abstract])) OR (Valsartan[Title/Abstract] AND Hydrochlorothiazide[Title/Abstract])) OR (Amlodipine/Benazepril[Title/Abstract])) OR (Amlodipine[Title/Abstract] AND Benazepril[Title/Abstract])) OR (Enalapril/Felodipine[Title/Abstract])) OR (Enalapril[Title/Abstract] AND Felodipine[Title/Abstract])) OR (Perindopril/Amlodipine[Title/Abstract])) OR (Perindopril[Title/Abstract] AND Amlodipine[Title/Abstract])) OR (Amlodipine/Olmesartan[Title/Abstract])) OR (Amlodipine[Title/Abstract] AND Olmesartan[Title/Abstract])) OR (Amlodipine/Valsartan[Title/Abstract])) OR (Amlodipine[Title/Abstract] AND Valsartan[Title/Abstract])) OR (Telmisartan/Amlodipine[Title/Abstract])) OR (Telmisartan[Title/Abstract] AND Amlodipine[Title/Abstract])) OR (Trandolapril/Verapamil[Title/Abstract])) OR (Trandolapril[Title/Abstract] AND Verapamil[Title/Abstract])) OR (Atenolol/Chlorthalidone[Title/Abstract])) OR (Atenolol[Title/Abstract] AND Chlorthalidone[Title/Abstract])) OR (Bisoprolol/Hydrochlorothiazide[Title/Abstract])) OR (Bisoprolol[Title/Abstract] AND Hydrochlorothiazide[Title/Abstract])) OR (Metoprolol succinate/Hydrochlorothiazide[Title/Abstract])) OR (Metoprolol succinate[Title/Abstract] AND Hydrochlorothiazide[Title/Abstract])) OR (Metoprolol tartrate/ Hydrochlorothiazide[Title/Abstract])) OR (Metoprolol tartrate[Title/Abstract] AND Hydrochlorothiazide[Title/Abstract])) OR (Nadolol/Bendroflumethiazide[Title/Abstract])) OR (Nadolol[Title/Abstract] AND Bendroflumethiazide[Title/Abstract])) OR (Propranolol/Hydrochlorothiazide[Title/Abstract])) OR (Propranolol/Hydrochlorothiazide[Title/Abstract])) OR (Aliskiren/amlodipine[Title/Abstract])) OR (Aliskiren[Title/Abstract] AND amlodipine[Title/Abstract])) OR (Aliskiren/ Hydrochlorothiazide[Title/Abstract])) OR (Aliskiren[Title/Abstract] AND Hydrochlorothiazide[Title/Abstract]) |
| #6 | #4 OR #5 |
| #7 | randomzied controlled trials[Publication Type] |
| #8 | ((Randomized) OR (RCT)) OR (Placebo) |
| #9 | #7 OR #8 |
| #10 | #3 AND #6 AND #9 |

Table S2. Consistency test for SBP

| Side | Direct  Coef. | Std. Err. | Indirect  Coef. | Std. Err. | Difference  Coef. | Std. Err. | P |
| --- | --- | --- | --- | --- | --- | --- | --- |
| A VS C | 8.679033 | 1.792252 | 6.198891 | 3.254969 | 2.480143 | 3.715774 | 0.504 |
| A VS F | ‐ .6999998 | 2.355378 | 3.172003 | 2.871994 | -2.472003 | 3.714317 | 0.506 |
| A VS K | 3.3 | 2.37028 | 8.244006 | 6.969073 | -4.944006 | 7.428633 | 0.506 |
| B VS D | 9.999947 | 2.637467 | 6.290587 | 2.676254 | 3.709361 | 3.757454 | 0.324 |
| B VS E | 5.276008 | 1.885179 | 7.546271 | 1.664786 | -2.270262 | 2.515541 | 0.367 |
| B VS G | 5.899726 | 1.684677 | 6.494897 | 2.209663 | -.5951717 | 2.783552 | 0.831 |
| B VS H | 5 | 2.318808 | 7.819702 | 632.773 | -2.819702 | 632.7746 | 0.996 |
| B VS I | 9.59999 | 2.470149 | 10.76117 | 2.835775 | -1.161182 | 3.760747 | 0.758 |
| B VS J | 6.291687 | 1.913095 | 3.236423 | 2.346493 | 3.055264 | 3.036408 | 0.314 |
| B VS L | 6.179124 | 1.64873 | 7.029118 | 4.077403 | -.8499941 | 4.397988 | 0.847 |
| B VS M | 8.19997 | 2.854174 | 8.068059 | 1.862621 | .1319113 | 3.408176 | 0.969 |
| B VS N | 5.182398 | 1.681679 | 5.625299 | 2.914197 | -.4429006 | 3.367149 | 0.895 |
| C VS F | -5.49996 | 2.24856 | -7.979696 | 2.958333 | 2.479736 | 3.715878 | 0.505 |
| D VS J | -1.899972 | 2.140623 | -5.609141 | 3.088145 | 3.70917 | 3.757516 | 0.324 |
| E VS F | -3.855609 | .9935995 | -1.221161 | 2.323476 | -2.634448 | 2.526325 | 0.297 |
| E VS N | -.9999397 | 2.557359 | -1.442974 | 2.190361 | .4430341 | 3.367153 | 0.895 |
| F VS G | -.2999447 | 2.392078 | 5.133902 | 1.921893 | -5.433847 | 3.068496 | 0.077 |
| F VS I | 7.500011 | 2.468201 | 6.338832 | 2.837427 | 1.16118 | 3.760708 | 0.757 |
| F VS K | 2.6 | 2.246911 | -2.344005 | 7.090616 | 4.944006 | 7.428634 | 0.506 |
| F VS M | 5.079379 | 1.50348 | 4.759295 | 2.759367 | .320084 | 3.142925 | 0.919 |
| G VS J | -5.699852 | 2.643359 | 1.700102 | 2.049752 | -7.399954 | 3.344967 | 0.027 |
| L VS M | 1.200253 | 3.715721 | 2.050579 | 2.353337 | -.8503254 | 4.398285 | 0.847 |

Tip : A:ACEI; B:ARB; C:Amlodipine/benazepril; D:Amlodipine/losartan; E:Amlodipine/valsartan; F:CCB; G:Candesartan/HCTZ; H:Irbesartan/HCTZ; I:Irbesartan/amlodipine; J:Losartan/HCTZ；K:Perindopril/amlodipine；L:Telmisartan/HCTZ；M:Telmisartan/amlodipine;N:Valsartan/HCTZ;

Table S3. Consistency test for DBP

| Side | Direct  Coef. | Std. Err. | Indirect  Coef. | Std. Err. | Difference  Coef. | Std. Err. | P |
| --- | --- | --- | --- | --- | --- | --- | --- |
| A VS C | 4.499431 | 1.158452 | 3.196497 | 58.73909 | 1.302935 | 58.75057 | 0.982 |
| B VS D | 8.499993 | 1.557196 | 4.797666 | 1.857148 | 3.702326 | 2.423602 | 0.127 |
| B VS E | 3.786675 | 1.515395 | 4.773535 | 1.155094 | -.9868598 | 1.90554 | 0.605 |
| B VS G | 2.612306 | 1.229763 | 3.759162 | 1.415289 | -1.146856 | 1.875074 | 0.541 |
| B VS H | 3 | 1.628996 | 4.335909 | 632.534 | -1.335909 | 632.5351 | 0.998 |
| B VS I | 4.899997 | 1.713635 | 4.619779 | 1.929232 | .2802186 | 2.580402 | 0.914 |
| B VS J | 3.440857 | 1.286979 | 2.522779 | 1.567374 | .9180781 | 2.029937 | 0.651 |
| B VS K | 2.760291 | 1.094883 | 6.76746 | 2.14468 | -4.007169 | 2.407896 | 0.096 |
| B VS L | 6.499996 | 1.702837 | 4.794524 | 1.235983 | 1.705472 | 2.104117 | 0.418 |
| B VS M | 2.556242 | 1.478813 | 2.500536 | 1.950773 | .0557063 | 2.448948 | 0.982 |
| C VS F | -3.099985 | 1.683867 | -4.357803 | 59.50015 | 1.257818 | 59.52386 | 0.983 |
| D VS J | -2.399984 | 1.542746 | -6.102234 | 1.869188 | 3.70225 | 2.42362 | 0.127 |
| E VS F | -2.501799 | .7628808 | -1.606605 | 1.68209 | -.8951935 | 1.846605 | 0.628 |
| E VS M | -1.899989 | 1.663767 | -1.844356 | 1.79701 | -.0556333 | 2.448951 | 0.982 |
| F VS G | -.0999958 | 1.561233 | 2.013358 | 1.444906 | -2.113354 | 2.127251 | 0.320 |
| F VS I | 2.600004 | 1.666427 | 2.880195 | 1.970137 | -.280191 | 2.580389 | 0.914 |
| F VS L | 3.56755 | 1.036815 | 2.627034 | 1.72104 | .9405154 | 2.009049 | 0.640 |
| G VS J | -2.599977 | 1.681387 | 1.765715 | 1.400502 | -4.365692 | 2.188256 | 0.046 |
| K VS L | -.5999685 | 1.870375 | 3.407383 | 1.516508 | -4.007351 | 2.407927 | 0.096 |

Tip: A :ACEI; B:ARB; C: Amlodipine/benazepril; D: Amlodipine/losartan; E:Amlodipine/valsartan; F:CCB; G:Candesartan/HCTZ; H:Irbesartan/HCTZ; I:Irbesartan/amlodipine; J:Losartan/HCTZ; K: Telmisartan/HCTZ; L: Telmisartan/amlodipine; M: Valsartan/HCTZ.

Table S4. Consistency test for blood pressure control rate

| Side | Direct  Coef. | Std. Err. | Indirect  Coef. | Std. Err. | Difference  Coef. | Std. Err. | P |
| --- | --- | --- | --- | --- | --- | --- | --- |
| A VS C | . | . | . | . | . | . | . |
| A VS F | .029853 | .393782 | -.0494831 | 67.53075 | .0793361 | 67.53189 | 0.999 |
| A VS K | .334793 | .3916875 | .1760754 | 135.02 | .1587176 | 135.0206 | 0.999 |
| B VS E | 1.118692 | .3947571 | 1.148206 | .3269749 | -.0295131 | .5125874 | 0.954 |
| B VS G | .5935681 | .2631078 | 1.065147 | .4178093 | -.4715788 | .4923562 | 0.338 |
| B VS H | .5162827 | .4020209 | 1.330046 | 634.9104 | -.8137632 | 634.9104 | 0.999 |
| B VS I | 1.055724 | .4152565 | 1.5484 | .5009413 | -.4926754 | .6506767 | 0.449 |
| B VS J | 1.401182 | .4174278 | .0930182 | .422276 | 1.308164 | .5937701 | 0.028 |
| B VS L | .6947735 | .3912042 | 1.3037 | .6637578 | -.6089261 | .7704642 | 0.429 |
| B VS M | 1.412269 | .5956365 | 1.27323 | .3272511 | .1390394 | .6796147 | 0.838 |
| B VS N | .6431878 | .3601113 | .2856601 | .54546 | .3575277 | .6536106 | 0.584 |
| D VS J | -.3890615 | .4509817 | 1.104847 | 631.2324 | -1.493908 | 631.2327 | 0.998 |
| E VS F | -.6666361 | .2029156 | -.8474267 | .4604235 | .1807906 | .5032713 | 0.719 |
| E VS N | -.7880533 | .4731648 | -.4305264 | .4509118 | -.3575269 | .6536102 | 0.584 |
| F VS G | -.0116959 | .5164352 | .4393182 | .3701205 | -.4510141 | .6353695 | 0.478 |
| F VS I | 1.024742 | .4235822 | .5320673 | .4939208 | .4926743 | .6506762 | 0.449 |
| F VS K | .3049404 | .3817333 | .4640458 | 134.9237 | -.1591054 | 134.9243 | 0.999 |
| F VS M | .9066386 | .2419062 | .6618531 | .5366524 | .2447855 | .5881471 | 0.677 |
| G VS J | -.4712796 | .3641006 | .8368821 | .4690339 | -1.308162 | .5937693 | 0.028 |
| L VS M | .1005418 | .5864106 | .7094695 | .4997397 | -.6089277 | .7704657 | 0.429 |

Tip: A:ACEI; B:ARB; C:Amlodipine/benazepril; D:Amlodipine/losartan; E:Amlodipine/valsartan; F:CCB; G: Candesartan/HCTZ; H: Irbesartan/HCTZ; I: Irbesartan/amlodipine; J:Losartan/HCTZ; K:Perindopril/amlodipine; L:Telmisartan/HCTZ; M: Telmisartan/amlodipine; N: Valsartan/HCTZ;

Table S5. Consistency test for DBP response rate

| Side | Direct  Coef. | Std. Err. | Indirect  Coef. | Std. Err. | Difference  Coef. | Std. Err. | P |
| --- | --- | --- | --- | --- | --- | --- | --- |
| A VS C | 1.585484 | .4563218 | .5152879 | 70.96656 | 1.070196 | 70.96802 | 0.988 |
| B VS D | 1.504078 | .6095628 | .7950589 | .6156576 | .7090189 | .8663722 | 0.413 |
| B VS E | .7024536 | .2941983 | 1.485759 | .6883284 | -.7833058 | .7485909 | 0.295 |
| B VS G | .6713221 | .3075656 | .8009828 | .4967189 | -.1296607 | .5837829 | 0.824 |
| B VS H | .7500216 | .4623326 | .4242284 | .4509491 | .3257932 | .6458378 | 0.614 |
| B VS I | .7017484 | .3130048 | .6721964 | 1.120529 | .029552 | 1.163413 | 0.980 |
| B VS K | .0821907 | .2800057 | -1.330336 | 445.3109 | 1.412527 | 445.3109 | 0.997 |
| C VS F | -.47394 | .4205725 | -1.543476 | 72.43283 | 1.069536 | 72.43404 | 0.988 |
| D VS H | -.3263871 | .5083574 | -1.035398 | .7015514 | .709011 | .8663727 | 0.413 |
| E VS F | -.5589117 | .291389 | .2244015 | .6895672 | -.7833133 | .748588 | 0.295 |
| F VS G | -.3678855 | .7113616 | .6272624 | .4670657 | -.9951479 | .8509908 | 0.242 |
| F VS J | .6354027 | .2645432 | .6649033 | 1.131932 | -.0295005 | 1.163414 | 0.980 |
| G VS H | -.4915938 | .4506137 | .2836867 | .4804441 | -.7752804 | .6586951 | 0.239 |
| I VS J | .348313 | 1.021063 | .318781 | .5576528 | .029532 | 1.16342 | 0.980 |

Tip：A: ACEI；B:ARB；C: Amlodipine/benazepril；D: Amlodipine/losartan；E: Amlodipine/valsartan；F: CCB；G: Candesartan/HCTZ；H:Losartan/HCTZ；I: Telmisartan/HCTZ；J: Telmisartan/amlodipine；K: Valsartan/HCTZ.

Table S6. League table on SBP

| Irbesartan/amlodipine | -0.59 (-5.53,4.35) | -2.00 (-6.15,2.15) | -1.93 (-7.03,3.17) | -3.55 (-7.30,0.20) | -3.81 (-8.34,0.73) | -3.99 (-8.17,0.20) | -4.84 (-10.28,0.59) | -4.83 (-9.18,-0.47) | -5.10 (-10.87,0.67) | -5.03 (-9.54,-0.53) | -7.00 (-10.55,-3.45) | -8.69 (-13.68,-3.71) | -10.10 (-13.65,-6.55) |
| --- | --- | --- | --- | --- | --- | --- | --- | --- | --- | --- | --- | --- | --- |
| 0.59 (-4.35,5.53) | Amlodipine/benazepril | -1.41 (-5.67,2.86) | -1.34 (-6.91,4.24) | -2.96 (-6.83,0.92) | -3.21 (-8.23,1.80) | -3.39 (-8.01,1.22) | -4.25 (-8.95,0.45) | -4.23 (-9.02,0.56) | -4.51 (-10.72,1.70) | -4.44 (-9.47,0.58) | -6.40 (-9.84,-2.97) | -8.10 (-11.12,-5.08) | -9.51 (-13.74,-5.27) |
| 2.00 (-2.15,6.15) | 1.41 (-2.86,5.67) | Telmisartan/amlodipine | 0.07 (-4.64,4.79) | -1.55 (-4.48,1.39) | -1.81 (-5.64,2.03) | -1.98 (-5.62,1.65) | -2.84 (-7.67,1.99) | -2.82 (-6.66,1.01) | -3.10 (-8.54,2.34) | -3.03 (-7.10,1.03) | -5.00 (-7.52,-2.47) | -6.69 (-11.01,-2.38) | -8.10 (-11.10,-5.11) |
| 1.93 (-3.17,7.03) | 1.34 (-4.24,6.91) | -0.07 (-4.79,4.64) | Amlodipine/losartan | -1.62 (-6.00,2.76) | -1.88 (-6.59,2.83) | -2.06 (-6.31,2.19) | -2.91 (-8.93,3.11) | -2.90 (-7.49,1.70) | -3.17 (-9.03,2.68) | -3.11 (-6.57,0.36) | -5.07 (-9.46,-0.67) | -6.76 (-12.38,-1.15) | -8.17 (-11.87,-4.48) |
| 3.55 (-0.20,7.30) | 2.96 (-0.92,6.83) | 1.55 (-1.39,4.48) | 1.62 (-2.76,6.00) | Amlodipine/valsartan | -0.26 (-3.94,3.42) | -0.44 (-3.61,2.74) | -1.29 (-5.78,3.20) | -1.28 (-4.45,1.89) | -1.55 (-6.70,3.60) | -1.48 (-5.15,2.18) | -3.45 (-5.25,-1.65) | -5.14 (-9.08,-1.21) | -6.55 (-8.98,-4.13) |
| 3.81 (-0.73,8.34) | 3.21 (-1.80,8.23) | 1.81 (-2.03,5.64) | 1.88 (-2.83,6.59) | 0.26 (-3.42,3.94) | Telmisartan/HCTZ | -0.18 (-4.01,3.66) | -1.03 (-6.54,4.47) | -1.02 (-5.01,2.98) | -1.29 (-6.71,4.12) | -1.23 (-5.31,2.86) | -3.19 (-6.85,0.47) | -4.88 (-9.95,0.18) | -6.29 (-9.24,-3.35) |
| 3.99 (-0.20,8.17) | 3.39 (-1.22,8.01) | 1.98 (-1.65,5.62) | 2.06 (-2.19,6.31) | 0.44 (-2.74,3.61) | 0.18 (-3.66,4.01) | Candesartan/HCTZ | -0.86 (-6.00,4.28) | -0.84 (-4.50,2.82) | -1.12 (-6.32,4.09) | -1.05 (-4.40,2.30) | -3.01 (-6.09,0.07) | -4.71 (-9.37,-0.05) | -6.12 (-8.65,-3.58) |
| 4.84 (-0.59,10.28) | 4.25 (-0.45,8.95) | 2.84 (-1.99,7.67) | 2.91 (-3.11,8.93) | 1.29 (-3.20,5.78) | 1.03 (-4.47,6.54) | 0.86 (-4.28,6.00) | Perindopril/amlodipine | 0.02 (-5.29,5.32) | -0.26 (-6.87,6.35) | -0.19 (-5.70,5.32) | -2.16 (-6.27,1.96) | -3.85 (-8.12,0.42) | -5.26 (-10.06,-0.46) |
| 4.83 (0.47,9.18) | 4.23 (-0.56,9.02) | 2.82 (-1.01,6.66) | 2.90 (-1.70,7.49) | 1.28 (-1.89,4.45) | 1.02 (-2.98,5.01) | 0.84 (-2.82,4.50) | -0.02 (-5.32,5.29) | Valsartan/HCTZ | -0.28 (-5.60,5.04) | -0.21 (-4.16,3.74) | -2.17 (-5.52,1.17) | -3.87 (-8.71,0.97) | -5.28 (-8.04,-2.51) |
| 5.10 (-0.67,10.87) | 4.51 (-1.70,10.72) | 3.10 (-2.34,8.54) | 3.17 (-2.68,9.03) | 1.55 (-3.60,6.70) | 1.29 (-4.12,6.71) | 1.12 (-4.09,6.32) | 0.26 (-6.35,6.87) | 0.28 (-5.04,5.60) | Irbesartan/HCTZ | 0.07 (-5.30,5.43) | -1.90 (-7.07,3.28) | -3.59 (-9.84,2.66) | -5.00 (-9.54,-0.46) |
| 5.03 (0.53,9.54) | 4.44 (-0.58,9.47) | 3.03 (-1.03,7.10) | 3.11 (-0.36,6.57) | 1.48 (-2.18,5.15) | 1.23 (-2.86,5.31) | 1.05 (-2.30,4.40) | 0.19 (-5.32,5.70) | 0.21 (-3.74,4.16) | -0.07 (-5.43,5.30) | Losartan/HCTZ | -1.96 (-5.63,1.70) | -3.66 (-8.73,1.41) | -5.07 (-7.92,-2.22) |
| 7.00 (3.45,10.55) | 6.40 (2.97,9.84) | 5.00 (2.47,7.52) | 5.07 (0.67,9.46) | 3.45 (1.65,5.25) | 3.19 (-0.47,6.85) | 3.01 (-0.07,6.09) | 2.16 (-1.96,6.27) | 2.17 (-1.17,5.52) | 1.90 (-3.28,7.07) | 1.96 (-1.70,5.63) | CCB | -1.69 (-5.19,1.80) | -3.10 (-5.58,-0.63) |
| 8.69 (3.71,13.68) | 8.10 (5.08,11.12) | 6.69 (2.38,11.01) | 6.76 (1.15,12.38) | 5.14 (1.21,9.08) | 4.88 (-0.18,9.95) | 4.71 (0.05,9.37) | 3.85 (-0.42,8.12) | 3.87 (-0.97,8.71) | 3.59 (-2.66,9.84) | 3.66 (-1.41,8.73) | 1.69 (-1.80,5.19) | ACEI | -1.41 (-5.70,2.88) |
| 10.10 (6.55,13.65) | 9.51 (5.27,13.74) | 8.10 (5.11,11.10) | 8.17 (4.48,11.87) | 6.55 (4.13,8.98) | 6.29 (3.35,9.24) | 6.12 (3.58,8.65) | 5.26 (0.46,10.06) | 5.28 (2.51,8.04) | 5.00 (0.46,9.54) | 5.07 (2.22,7.92) | 3.10 (0.63,5.58) | 1.41 (-2.88,5.70) | ARB |

Table S7 . League table on DBP

| Amlodipine/losartan | -1.58 (-4.67,1.50) | -1.81 (-6.22,2.61) | -2.19 (-5.62,1.24) | -2.56 (-5.54,0.42) | -3.37 (-6.52,-0.22) | -3.87 (-6.74,-1.00) | -3.97 (-7.99,0.05) | -3.90 (-6.33,-1.47) | -4.44 (-7.75,-1.13) | -4.91 (-7.85,-1.97) | -6.30 (-11.27,-1.34) | -6.97 (-9.41,-4.53) |
| --- | --- | --- | --- | --- | --- | --- | --- | --- | --- | --- | --- | --- |
| 1.58 (-1.50,4.67) | Telmisartan/amlodipine | -0.22 (-3.94,3.49) | -0.61 (-3.39,2.18) | -0.97 (-3.01,1.06) | -1.79 (-4.20,0.63) | -2.28 (-4.69,0.12) | -2.39 (-6.12,1.35) | -2.32 (-4.97,0.34) | -2.86 (-5.62,-0.09) | -3.32 (-5.03,-1.61) | -4.72 (-9.07,-0.37) | -5.39 (-7.32,-3.45) |
| 1.81 (-2.61,6.22) | 0.22 (-3.49,3.94) | Amlodipine/benazepril | -0.39 (-4.48,3.70) | -0.75 (-4.31,2.80) | -1.57 (-5.67,2.54) | -2.06 (-5.96,1.83) | -2.17 (-7.07,2.73) | -2.10 (-6.21,2.02) | -2.64 (-6.76,1.49) | -3.10 (-6.40,0.20) | -4.50 (-6.77,-2.23) | -5.17 (-8.89,-1.45) |
| 2.19 (-1.24,5.62) | 0.61 (-2.18,3.39) | 0.39 (-3.70,4.48) | Irbesartan/amlodipine | -0.37 (-2.99,2.25) | -1.18 (-4.25,1.90) | -1.68 (-4.54,1.18) | -1.78 (-5.80,2.24) | -1.71 (-4.76,1.34) | -2.25 (-5.42,0.92) | -2.71 (-5.13,-0.29) | -4.11 (-8.79,0.57) | -4.78 (-7.22,-2.34) |
| 2.56 (-0.42,5.54) | 0.97 (-1.06,3.01) | 0.75 (-2.80,4.31) | 0.37 (-2.25,2.99) | Amlodipine/valsartan | -0.81 (-3.35,1.73) | -1.31 (-3.54,0.92) | -1.41 (-5.06,2.24) | -1.34 (-3.87,1.19) | -1.88 (-4.22,0.45) | -2.35 (-3.68,-1.01) | -3.74 (-7.97,0.48) | -4.41 (-6.19,-2.64) |
| 3.37 (0.22,6.52) | 1.79 (-0.63,4.20) | 1.57 (-2.54,5.67) | 1.18 (-1.90,4.25) | 0.81 (-1.73,3.35) | Telmisartan/HCTZ | -0.50 (-3.12,2.12) | -0.60 (-4.37,3.17) | -0.53 (-3.28,2.21) | -1.07 (-4.03,1.89) | -1.54 (-3.98,0.91) | -2.93 (-7.63,1.76) | -3.60 (-5.60,-1.60) |
| 3.87 (1.00,6.74) | 2.28 (-0.12,4.69) | 2.06 (-1.83,5.96) | 1.68 (-1.18,4.54) | 1.31 (-0.92,3.54) | 0.50 (-2.12,3.12) | Candesartan/HCTZ | -0.10 (-3.76,3.55) | -0.03 (-2.28,2.21) | -0.57 (-3.34,2.20) | -1.04 (-3.11,1.04) | -2.44 (-6.95,2.08) | -3.10 (-4.89,-1.32) |
| 3.97 (-0.05,7.99) | 2.39 (-1.35,6.12) | 2.17 (-2.73,7.07) | 1.78 (-2.24,5.80) | 1.41 (-2.24,5.06) | 0.60 (-3.17,4.37) | 0.10 (-3.55,3.76) | Irbesartan/HCTZ | 0.07 (-3.65,3.79) | -0.47 (-4.38,3.44) | -0.93 (-4.56,2.69) | -2.33 (-7.73,3.07) | -3.00 (-6.19,0.19) |
| 3.90 (1.47,6.33) | 2.32 (-0.34,4.97) | 2.10 (-2.02,6.21) | 1.71 (-1.34,4.76) | 1.34 (-1.19,3.87) | 0.53 (-2.21,3.28) | 0.03 (-2.21,2.28) | -0.07 (-3.79,3.65) | Losartan/HCTZ | -0.54 (-3.46,2.38) | -1.00 (-3.47,1.46) | -2.40 (-7.10,2.30) | -3.07 (-4.97,-1.17) |
| 4.44 (1.13,7.75) | 2.86 (0.09,5.62) | 2.64 (-1.49,6.76) | 2.25 (-0.92,5.42) | 1.88 (-0.45,4.22) | 1.07 (-1.89,4.03) | 0.57 (-2.20,3.34) | 0.47 (-3.44,4.38) | 0.54 (-2.38,3.46) | Valsartan/HCTZ | -0.46 (-2.94,2.01) | -1.86 (-6.57,2.85) | -2.53 (-4.79,-0.27) |
| 4.91 (1.97,7.85) | 3.32 (1.61,5.03) | 3.10 (-0.20,6.40) | 2.71 (0.29,5.13) | 2.35 (1.01,3.68) | 1.54 (-0.91,3.98) | 1.04 (-1.04,3.11) | 0.93 (-2.69,4.56) | 1.00 (-1.46,3.47) | 0.46 (-2.01,2.94) | CCB | -1.40 (-5.40,2.61) | -2.07 (-3.78,-0.35) |
| 6.30 (1.34,11.27) | 4.72 (0.37,9.07) | 4.50 (2.23,6.77) | 4.11 (-0.57,8.79) | 3.74 (-0.48,7.97) | 2.93 (-1.76,7.63) | 2.44 (-2.08,6.95) | 2.33 (-3.07,7.73) | 2.40 (-2.30,7.10) | 1.86 (-2.85,6.57) | 1.40 (-2.61,5.40) | ACEI | -0.67 (-5.03,3.69) |
| 6.97 (4.53,9.41) | 5.39 (3.45,7.32) | 5.17 (1.45,8.89) | 4.78 (2.34,7.22) | 4.41 (2.64,6.19) | 3.60 (1.60,5.60) | 3.10 (1.32,4.89) | 3.00 (-0.19,6.19) | 3.07 (1.17,4.97) | 2.53 (0.27,4.79) | 2.07 (0.35,3.78) | 0.67 (-3.69,5.03) | ARB |

Table S8. League table on BP control rate

| Telmisartan/amlodipine | 1.14 (0.32,4.05) | 0.95 (0.47,1.95) | 0.84 (0.50,1.42) | 0.86 (0.26,2.88) | 0.63 (0.30,1.33) | 0.58 (0.26,1.33) | 0.57 (0.24,1.35) | 0.56 (0.29,1.08) | 0.45 (0.17,1.19) | 0.46 (0.22,0.97) | 0.41 (0.17,0.99) | 0.42 (0.28,0.64) | 0.27 (0.16,0.47) |
| --- | --- | --- | --- | --- | --- | --- | --- | --- | --- | --- | --- | --- | --- |
| 0.87 (0.25,3.10) | Amlodipine/benazepril | 0.83 (0.22,3.20) | 0.74 (0.21,2.56) | 0.75 (0.14,4.00) | 0.56 (0.14,2.26) | 0.51 (0.12,2.11) | 0.50 (0.15,1.64) | 0.49 (0.13,1.85) | 0.40 (0.09,1.79) | 0.41 (0.10,1.58) | 0.36 (0.14,0.89) | 0.37 (0.11,1.22) | 0.24 (0.07,0.86) |
| 1.05 (0.51,2.15) | 1.20 (0.31,4.60) | Irbesartan/amlodipine | 0.88 (0.45,1.72) | 0.90 (0.26,3.14) | 0.67 (0.28,1.59) | 0.61 (0.25,1.47) | 0.60 (0.23,1.59) | 0.59 (0.28,1.22) | 0.48 (0.18,1.30) | 0.49 (0.22,1.09) | 0.43 (0.16,1.16) | 0.44 (0.24,0.82) | 0.28 (0.15,0.53) |
| 1.19 (0.71,2.00) | 1.36 (0.39,4.70) | 1.13 (0.58,2.20) | Amlodipine/valsartan | 1.02 (0.32,3.30) | 0.75 (0.35,1.60) | 0.69 (0.32,1.50) | 0.68 (0.30,1.55) | 0.67 (0.37,1.21) | 0.54 (0.22,1.35) | 0.55 (0.30,1.03) | 0.49 (0.21,1.13) | 0.50 (0.35,0.71) | 0.32 (0.20,0.51) |
| 1.16 (0.35,3.90) | 1.33 (0.25,7.07) | 1.11 (0.32,3.86) | 0.98 (0.30,3.17) | Amlodipine/losartan | 0.74 (0.21,2.62) | 0.68 (0.28,1.64) | 0.67 (0.17,2.67) | 0.65 (0.22,1.93) | 0.53 (0.14,2.03) | 0.54 (0.16,1.84) | 0.48 (0.12,1.94) | 0.49 (0.15,1.58) | 0.32 (0.11,0.94) |
| 1.58 (0.75,3.30) | 1.80 (0.44,7.32) | 1.50 (0.63,3.59) | 1.33 (0.62,2.82) | 1.36 (0.38,4.81) | Telmisartan/HCTZ | 0.92 (0.37,2.28) | 0.90 (0.32,2.58) | 0.89 (0.41,1.92) | 0.72 (0.26,1.99) | 0.73 (0.31,1.71) | 0.65 (0.22,1.88) | 0.66 (0.32,1.39) | 0.43 (0.22,0.82) |
| 1.72 (0.75,3.91) | 1.96 (0.47,8.11) | 1.64 (0.68,3.94) | 1.45 (0.67,3.14) | 1.48 (0.61,3.57) | 1.09 (0.44,2.70) | Losartan/HCTZ | 0.98 (0.34,2.87) | 0.96 (0.51,1.80) | 0.78 (0.28,2.16) | 0.80 (0.34,1.87) | 0.70 (0.24,2.09) | 0.72 (0.34,1.56) | 0.47 (0.24,0.89) |
| 1.75 (0.74,4.12) | 2.00 (0.61,6.57) | 1.67 (0.63,4.41) | 1.47 (0.64,3.37) | 1.50 (0.37,6.04) | 1.11 (0.39,3.17) | 1.02 (0.35,2.98) | Perindopril/amlodipine | 0.98 (0.38,2.52) | 0.79 (0.24,2.60) | 0.81 (0.30,2.19) | 0.72 (0.33,1.54) | 0.74 (0.35,1.56) | 0.47 (0.20,1.15) |
| 1.78 (0.92,3.43) | 2.04 (0.54,7.65) | 1.70 (0.82,3.52) | 1.50 (0.83,2.72) | 1.53 (0.52,4.53) | 1.13 (0.52,2.45) | 1.04 (0.55,1.94) | 1.02 (0.40,2.62) | Candesartan/HCTZ | 0.81 (0.33,1.99) | 0.83 (0.41,1.67) | 0.73 (0.28,1.91) | 0.75 (0.42,1.34) | 0.48 (0.31,0.75) |
| 2.20 (0.84,5.75) | 2.51 (0.56,11.33) | 2.10 (0.77,5.71) | 1.85 (0.74,4.64) | 1.89 (0.49,7.28) | 1.40 (0.50,3.89) | 1.28 (0.46,3.54) | 1.26 (0.39,4.11) | 1.24 (0.50,3.04) | Telmisartan/HCTZ | 1.02 (0.39,2.70) | 0.90 (0.27,2.99) | 0.93 (0.37,2.32) | 0.60 (0.27,1.31) |
| 2.16 (1.04,4.49) | 2.46 (0.63,9.61) | 2.06 (0.92,4.60) | 1.82 (0.97,3.39) | 1.85 (0.54,6.33) | 1.37 (0.58,3.21) | 1.26 (0.54,2.95) | 1.23 (0.46,3.34) | 1.21 (0.60,2.45) | 0.98 (0.37,2.59) | Valsartan/HCTZ | 0.88 (0.32,2.43) | 0.91 (0.47,1.75) | 0.58 (0.33,1.03) |
| 2.44 (1.02,5.88) | 2.79 (1.13,6.93) | 2.33 (0.86,6.27) | 2.06 (0.88,4.81) | 2.10 (0.52,8.54) | 1.55 (0.53,4.51) | 1.42 (0.48,4.23) | 1.40 (0.65,3.01) | 1.37 (0.52,3.59) | 1.11 (0.33,3.69) | 1.13 (0.41,3.12) | ACEI | 1.03 (0.48,2.23) | 0.66 (0.27,1.64) |
| 2.37 (1.56,3.61) | 2.71 (0.82,8.93) | 2.26 (1.21,4.21) | 2.00 (1.41,2.84) | 2.04 (0.63,6.58) | 1.50 (0.72,3.15) | 1.38 (0.64,2.98) | 1.36 (0.64,2.87) | 1.33 (0.75,2.37) | 1.08 (0.43,2.70) | 1.10 (0.57,2.12) | 0.97 (0.45,2.10) | CCB | 0.64 (0.40,1.03) |
| 3.69 (2.13,6.39) | 4.21 (1.17,15.19) | 3.51 (1.89,6.52) | 3.11 (1.94,4.97) | 3.17 (1.06,9.46) | 2.34 (1.22,4.50) | 2.15 (1.13,4.09) | 2.11 (0.87,5.11) | 2.07 (1.34,3.21) | 1.68 (0.76,3.68) | 1.71 (0.97,3.02) | 1.51 (0.61,3.73) | 1.55 (0.97,2.49) | ARB |

Table S9. League table on DBP response rate

| Amlodipine/losartan | 0.88 (0.27,2.80) | 0.72 (0.27,1.94) | 0.75 (0.19,2.88) | 0.64 (0.26,1.60) | 0.64 (0.23,1.77) | 0.57 (0.25,1.26) | 0.46 (0.16,1.35) | 0.34 (0.13,0.94) | 0.32 (0.14,0.73) | 0.15 (0.03,0.77) |
| --- | --- | --- | --- | --- | --- | --- | --- | --- | --- | --- |
| 1.14 (0.36,3.64) | Telmisartan/amlodipine | 0.82 (0.40,1.67) | 0.85 (0.32,2.23) | 0.73 (0.30,1.81) | 0.73 (0.28,1.87) | 0.65 (0.24,1.76) | 0.53 (0.32,0.87) | 0.39 (0.15,1.05) | 0.36 (0.16,0.82) | 0.17 (0.05,0.65) |
| 1.39 (0.52,3.74) | 1.22 (0.60,2.48) | Amlodipine/valsartan | 1.04 (0.39,2.76) | 0.89 (0.45,1.78) | 0.88 (0.41,1.90) | 0.79 (0.35,1.75) | 0.64 (0.38,1.10) | 0.48 (0.22,1.03) | 0.44 (0.26,0.75) | 0.21 (0.06,0.80) |
| 1.34 (0.35,5.18) | 1.18 (0.45,3.08) | 0.97 (0.36,2.58) | Amlodipine/benazepril | 0.86 (0.28,2.68) | 0.85 (0.26,2.80) | 0.76 (0.22,2.56) | 0.62 (0.27,1.42) | 0.46 (0.14,1.53) | 0.42 (0.15,1.24) | 0.20 (0.08,0.50) |
| 1.56 (0.62,3.88) | 1.36 (0.55,3.37) | 1.12 (0.56,2.24) | 1.16 (0.37,3.61) | Candesartan/HCTZ | 0.99 (0.46,2.11) | 0.88 (0.46,1.68) | 0.72 (0.33,1.58) | 0.53 (0.26,1.12) | 0.49 (0.30,0.81) | 0.24 (0.06,1.01) |
| 1.57 (0.57,4.37) | 1.38 (0.53,3.56) | 1.13 (0.53,2.44) | 1.17 (0.36,3.85) | 1.01 (0.47,2.16) | Telmisartan/HCTZ | 0.89 (0.38,2.07) | 0.73 (0.31,1.72) | 0.54 (0.24,1.20) | 0.50 (0.28,0.89) | 0.24 (0.05,1.06) |
| 1.77 (0.79,3.94) | 1.55 (0.57,4.22) | 1.27 (0.57,2.84) | 1.32 (0.39,4.45) | 1.14 (0.60,2.17) | 1.12 (0.48,2.62) | Losartan/HCTZ | 0.82 (0.34,2.01) | 0.61 (0.27,1.39) | 0.56 (0.30,1.04) | 0.27 (0.06,1.22) |
| 2.15 (0.74,6.28) | 1.89 (1.15,3.10) | 1.55 (0.91,2.64) | 1.61 (0.70,3.66) | 1.38 (0.63,3.02) | 1.37 (0.58,3.23) | 1.22 (0.50,2.98) | CCB | 0.74 (0.31,1.78) | 0.68 (0.34,1.35) | 0.33 (0.10,1.11) |
| 2.92 (1.07,7.97) | 2.56 (0.95,6.85) | 2.10 (0.98,4.52) | 2.17 (0.65,7.25) | 1.87 (0.89,3.92) | 1.85 (0.83,4.12) | 1.65 (0.72,3.77) | 1.35 (0.56,3.26) | Valsartan/HCTZ | 0.92 (0.53,1.59) | 0.45 (0.10,2.00) |
| 3.16 (1.36,7.35) | 2.77 (1.22,6.29) | 2.28 (1.33,3.90) | 2.36 (0.81,6.90) | 2.03 (1.24,3.34) | 2.01 (1.12,3.60) | 1.79 (0.97,3.32) | 1.47 (0.74,2.92) | 1.09 (0.63,1.88) | ARB | 0.48 (0.12,1.95) |
| 6.55 (1.29,33.09) | 5.74 (1.54,21.35) | 4.72 (1.25,17.78) | 4.88 (2.00,11.94) | 4.21 (0.99,17.83) | 4.16 (0.94,18.42) | 3.70 (0.82,16.76) | 3.04 (0.90,10.25) | 2.25 (0.50,10.07) | 2.07 (0.51,8.36) | ACEI |
